# Effects of graphene oxide on grape growth, rhizosphere microbial diversity, and berry quality

**DOI:** 10.3389/fpls.2026.1941614

**Published:** 2026-08-26

**Authors:** Xiaokang Chen, JiaSheng Yang, Suhui Feng, Jiao Sun, Jianxin Hao, Penghao Li, Huina Gao, Guoxiang Li, Jingwei Li, Jun Qiao, Jianguo Zhao

**Affiliations:** 1 Engineering Research Center of Coal-based Ecological Carbon Sequestration Technology of the Ministry of Education, Shanxi Datong University, Datong, China; 2 Key Laboratory of Graphene Forestry Application of National Forest and Grass Administration, Shanxi Datong University, Datong, China; 3 School of Chemistry and Chemical Engineering, Shanxi Datong University, Datong, China; 4 Yunnan Dikangyuan Biotechnology Co., Ltd., Kunming, China

**Keywords:** grape, graphene oxide, microbial diversity, plant growth, quality

## Abstract

Grape (*Vitis vinifera* L.) is an economically important fruit crop worldwide, with berry size and quality determining yield and market value. Graphene oxide (GO), a carbon nanomaterial with high specific surface area and abundant oxygen-containing functional groups, exhibits excellent fertilizer adsorption, holding promise for agricultural applications. This study systematically evaluated the effects of exogenous GO application on growth, rhizosphere microecology, and berry quality in ‘Crimson Seedless’ grape. The results showed that, compared with the control, treatment with 25 mg/L GO significantly promoted the plant height, stem diameter, and biomass accumulation of grape seedlings, and enhanced photosynthetic capacity. Rhizosphere soil microbial diversity analysis revealed that GO treatment increased the abundance and diversity of both bacteria and fungi. Further species composition and Linear discriminant analysis Effect Size (LEfSe) analysis indicated that GO treatment significantly enriched potentially beneficial microorganisms in the grape rhizosphere, including *Bacillus*, *Sphingomonas*, *RB41*, and *Mortierella*. Soil physicochemical property measurements demonstrated that, compared with the control, GO significantly increased the contents of soil organic matter, total nitrogen, ammonium nitrogen, available potassium, and humic acid by 26.29%, 65.57%, 26.02%, 181.79%, and 14.05%, respectively, while reducing total phosphorus, total potassium, and available phosphorus. Regarding fruit quality, GO significantly increased berry weight, firmness, shape index, and contents of total sugar, vitamin C, soluble solids, tannins, anthocyanins, calcium and 23 Amino acid in berries compared with both the control and Bayer fertilizers (BR, Bayer AG, Cat: Dabs-1L) treatment. Collectively, these findings elucidate GO-mediated growth promotion and fruit quality regulation in grape, providing a theoretical and practical basis for broader agricultural GO application.

## 1 Introduction

Graphene, as a type of nanomaterial, possesses unique physical and electrical properties, and is increasingly being applied across fields such as biology, electronics, and medicine (Georgakilas et al., 2015; Mukherjee et al., 2016; Jiao et al., 2025). As an important derivative of graphene, graphene oxide (GO) is rich in oxygen-containing functional groups and possesses high electrical conductivity, large specific surface area, and excellent surface activity, demonstrating broad application prospects in fields such as agriculture (Avouris, 2010; Chandel et al., 2022). It retains the unique two-dimensional layered structure of graphene, which consists of sp²-hybridized carbon atoms arranged in a honeycomb lattice to form a planar film (Du et al., 2015). The surface of GO contains abundant hydrophilic oxygen-containing functional groups, such as carboxyl, carbonyl, epoxy, and hydroxyl groups, enabling it to disperse stably in water. In aqueous environments, it undergoes behaviors such as adsorption, agglomeration, and dispersion, resulting in an increased interlayer spacing (Luo et al., 2017). These oxygen-containing functional groups can effectively adsorb metal ions through electrostatic interactions, while the larger interlayer spacing also provides more adsorption sites for metal ions. Compared to the graphene, GO exhibits more significant surface activity, is easier to modify, and possesses better biocompatibility, making it more amenable to functionalization and endowed with superior overall performance (Feng et al., 2011). Based on these characteristics, GO has been widely applied in various interdisciplinary fields, including biomedicine, fluorescence detection, biosensors, antibacterial materials, electronics, and agriculture (Zhang et al., 2010; Miao et al., 2026).

Grape (*Vitis vinifera* L.), belonging to the genus Vitis in the family Vitaceae, is a tall climbing vine and one of the most economically important fruit crops globally, with a long history of cultivation. Among numerous varieties, Crimson Seedless grape stands out for its extended shelf life and convenience, making it highly popular among consumers. It is distinguished by its bright red color, firm flesh texture, and sweet flavor, establishing it as a mainstream variety in international trade. As a high-value grape cultivar, enhancing the yield and quality of Crimson Seedless can significantly increase farm income and support sustainable agricultural development.

Despite its notable economic advantages, the cultivation of Crimson Seedless faces multiple challenges. Environmental stresses such as drought, pests and diseases, soil nutrient depletion, and climate variability can adversely affect plant growth, yield, and fruit quality. Traditional agricultural inputs are often inefficient, leading to environmental pollution and resource wastage. For example, growers have attempted to improve grape quality and yield through the application of various fertilizers, such as Bayer fertilizers and Shiqinhua liquid fertilizers (SL, Yunnan Dikangyuan Biotechnology Co., Ltd); however, these practices have introduced significant environmental problems. In this context, the application of nanomaterials in agricultural production has emerged as a transformative approach to enhance productivity and sustainability. Graphene, as a novel carbon material, leverages its unique physicochemical properties to improve nutrient delivery, stress resistance, and overall plant performance (Chen et al., 2022; Cao et al., 2024; Yan et al., 2024; Qiao et al., 2025a; Ge et al., 2026).

In recent years, researchers both domestically and internationally have conducted extensive studies on the application of graphene in agriculture and forestry. Studies have shown that graphene promotes the growth of various plants, including crops, flowers, trees, and medicinal plants. This promotive effect is mainly manifested in two aspects: first, influencing seed germination, root development, and seedling growth; and second, enhancing plant stress resistance (Wang et al., 2026; Chen et al., 2022; Zhao et al., 2026).

Regarding seed germination, graphene can penetrate the seed coat, promote water absorption, thereby accelerating germination speed and increasing germination rates. However, the promotive effect varies depending on plant species and graphene concentration (He et al., 2018; Yan et al., 2024). In terms of root growth, an appropriate amount of graphene can attach to the root tip meristematic and elongation zones, with its accumulation tendency increasing with higher concentrations. Nano-sized graphene (<100 nm) can also enter cells via endocytosis and interact with biomolecules, thereby promoting root development (Chen et al., 2022; Xie et al., 2020; Dąbrowski et al., 2023). Furthermore, graphene positively regulates the physiological and molecular response mechanisms of plants, enhancing crop tolerance to abiotic stresses such as drought, salinity, alkalinity, and heavy metals. Its effects are associated with strengthening antioxidant defenses, mitigating oxidative damage, and inducing the expression of stress-resistant genes (Li et al., 2021; Chen et al., 2021).

Despite the outstanding performance of graphene in promoting plant growth and enhancing stress resistance, its impact on fruit quality remains unclear. Therefore, this study aims to further elucidate the mechanisms by which graphene regulates grape growth and quality formation by investigating the effects of exogenous graphene on grape growth, fruit quality, and rhizosphere microecology.

## 2 Materials and methods

### 2.1 GO preparation and characterization

This study utilized a custom-built electrochemical setup to produce functionalized GO, where graphite served dual roles as both anode and cathode and distilled water acted as the electrolyte. A high-frequency pulsed current drove the electrolysis and oxidation of the graphite electrode, resulting in functionalized graphene. The resulting electrochemical field enabled external electrolyte ions to insert themselves between the graphite layers—similar to liquid-phase exfoliation. This field-driven mechanism forced electrolyte molecules into the graphite cathode, widening the interlayer spacing and reducing van der Waals forces. As a result, the desired functionalized GO was obtained through electrochemical delamination of graphite without any oxidation. The prepared graphene product is a black, homogeneous liquid with a solid content of 5 g·L^-^¹, a pH of 4, a specific surface area of ≥500 m²·g^-^¹, a purity of 95%, and an electrical conductivity (at 20 °C) of ≥7 S·cm^-^¹.The morphology of GO was characterized by scanning electron microscopy (SEM, TESCAN MAIA 3 LMH) and Raman Spectroscopy (HORIBA, LabRAM HR Evolution) with a 532 nm excitation laser (Renishaw inVia Qontor). Fourier-transform infrared (FTIR) spectroscopy was conducted using a Thermo Scientific Summit X spectrometer, and ultraviolet-visible (UV-Vis) absorption spectra were measured over the range of 200 to 800 nm with a SHIMADZU UV-3600 spectrophotometer.

### 2.2 Plant cultivation and GO exposure

The grape cultivar ‘Crimson Seedless’ seedlings were grown in a greenhouse at a controlled temperature of 25°C with a 16h light/8h dark photoperiod. At the two year growth stage, the seedlings were treated with graphene at various concentrations (0, 10, 25, 50, and 100 mg/L). Each treatment consisted of ten pots (one grape seedling per pot). Each pot contained 2 kg of soil, and 100 mL of the graphene solution was applied every ten days over a month period, with water serving as the control. After the treatment period, data were collected on plant height, stem diameter, and grape biomass.

### 2.3 Field planting experiment

Seedlings of the grape cultivar ‘Crimson Seedless’ were planted in Binchuan County, Dali Bai Autonomous Prefecture, Yunnan Province, located between 25°32′–26°12′ N latitude and 100°16′–100°59′ E longitude. The experiment was divided into two groups: CK (control) and GO25. Each treatment consisted of ten grape seedlings. Three-year-old grape seedlings were selected. At planting, each planting hole was irrigated with 100 mL of the respective GO solution (25 mg/L). This treatment was repeated at 10-day intervals for a total of three applications. After three months, for the rhizosphere soil collection, 3 grape plants were randomly selected from each treatment group, and then the three independent rhizosphere soil samples were carefully collected. Then, the collected samples were flash-frozen in liquid nitrogen and stored at −80 °C for subsequent microbiome sequencing.

### 2.4 Plant photosynthesis analysis

Photosynthetic parameters were measured on a clear, windless morning (9:00–11:30 AM) in late July at the experimental site in Binchuan County, Dali Bai Autonomous Prefecture, Yunnan Province, China. A CIRAS-3 Portable Photosynthesis System (PP Systems, USA) was used for all measurements. temperature at 25 °C, CO_2_ concentration at 390 µmol mol^-^¹, an open gas path system, and photosynthetically active radiation (PAR) set to 1,200 µmol m^-^² s^-^¹. For each treatment, three plants of comparable growth vigor were selected. From each plant, fully expanded functional leaves at the middle position of the current-year shoot (4th–6th leaves from the apex) were chosen for measurement. Within each treatment, leaves at the same node position were tagged and measured. Three leaves were measured per plant, and for each leaf, three stable readings were recorded. The average of these readings was used as the final value for that leaf. The following photosynthetic parameters were determined: intercellular CO_2_ concentration (Ci), transpiration rate (Tr), net photosynthetic rate (Pn), and stomatal conductance (Gs).

### 2.5 Determination of agronomic traits of grape

To assess plant growth, three key parameters were recorded. Plant height was defined as the length from the cotyledon node to the apical meristem and measured using a 2-meter ruler. Stem diameter was determined at the midpoint above the root–stem junction with a vernier caliper. grape biomass was quantified using an analytical balance (Shanghai Liangping, MA200 D1(M)00089).

### 2.6 Microbiome sequencing and analysis

Total DNA was extracted from 0.25 g of soil sample using a DNA extraction kit (Tiangen, DP812). The purity and concentration of the extracted DNA were measured using a NanoDrop microspectrophotometer (Thermo Fisher). The DNA was then diluted with sterile water to 1 ng/mL and used as a template for subsequent PCR amplification. For the amplification of the V3–V4 hypervariable region of the prokaryotic 16S rRNA gene, primers 338F (5’-ACTCCTACGGGAGGCAGCAG-3’) and 806R (5’-GGACTACHVGGGTWTCTAAT-3’) were employed, while the fungal ITS region was targeted using primers ITS5-F (5’-GGAAGTAAAAGTCGTAACAAGG-3’) and ITS2R (5’-GCTGCGTTCTTCATCGATGC-3’). The PCR reaction was performed in a 50 μL system under the following program: initial denaturation at 95 °C for 5 min, followed by 30 cycles of 95 °C for 1 min, 60 °C for 1 min, and 72 °C for 1 min, with a final extension at 72 °C for 7 min. The amplified products were purified using AMPure XP magnetic beads, after which a library was constructed and sequencing was carried out on the Illumina NovaSeq 6000 platform [79,80]. The resulting clean tags were clustered into operational taxonomic units (OTUs) at a 97% similarity threshold using USEARCH software (v9.2.64). The bacterial and fungal OTUs were taxonomically annotated using the RDP classifier (v2.2) against the SILVA database (version 132) and the UNITE database (version 8.0), respectively, with a confidence threshold of 0.8. Based on this, community composition analysis was conducted at all taxonomic levels from phylum to species.

### 2.7 Diversity and redundancy analysis of physicochemical properties

A Venn diagram illustrating inter-group relationships was generated using the VennDiagram package (v1.6.16) in R software. Diversity indices, including Chao1 and Shannon, were computed with QIIME (v1.9.1). To detect significant differences between the CK and treatment groups at the genus level, LEfSe analysis was employed. The Chao1 index reflects the number of OTUs detected through sequencing, whereas the Shannon index provides a comprehensive measure of species richness. Notably, higher values of these four indices correspond to greater microbial diversity. Principal component analysis (PCA), analysis of similarity (ANOSIM), and redundancy analysis (RDA) were conducted using the Vegan package (v2.5.3) in R. Additionally, Shannon index curves and rank abundance curves were visualized with the ggplot2 package (v2.2.1) in R language.

### 2.8 Determination of physicochemical properties of grape rhizosphere soil

After three months, for the rhizosphere soil collection, 3 grape plants were randomly selected from each treatment group, and then the three independent rhizosphere soil samples were carefully collected for soil physicochemical property analysis. To characterize soil physicochemical properties, we followed standard protocols. To begin, we homogenized a 10 g rhizosphere soil aliquot in 25 mL of deionized water, shaking the mixture for 60 seconds before letting it settle for 30 minutes. We then measured the pH using a calibrated Thermo Orion pH meter (Waltham, MA, USA). In parallel, a TPY-8A rapid analyzer allowed us to quantify bioavailable nutrients, specifically ammonium nitrogen (NH_4_^+^-N), available potassium (AK), and available phosphorus (AP), following the method described by Liu et al. (2020).

For soil organic matter (SOM), we employed a dichromate oxidation approach. A glucose standard (1.376 g, 0.5%-C solution) was first prepared in a 100 mL volumetric flask using concentrated H_2_SO_4_. Subsequently, we reacted 1 g soil samples with 10 mL of 0.4 M K_2_Cr_2_O_7_ and 10 mL of concentrated H_2_SO_4_ under vigorous shaking for 20 minutes. After dilution and settling, we measured the absorbance of the supernatant at 590 nm using a soil nutrient meter. Total nitrogen (TN) was assessed via the Kjeldahl method (HJ 717-2014; MEP, 2014). Meanwhile, total phosphorus (TP), total potassium (TK), and humic acid (HA) were measured according to Chinese agricultural standards NY/T 88-1988, NY/T 87-1988, and GB/T 45891-2025, respectively (Bao, 2005). Certified reference materials served as quality controls throughout all analyses.

### 2.9 Determination of grape fruit quality

During grape quality assessment, BR fertilizer and graphene-free fertilizer were used as controls. The total sugar content of grape berries was determined using the anthrone-sulfuric method (Leyva et al., 2008), and the soluble solids content was measured by the digital pocket refractometer for fruit (PAL-1, ATAGO). The contents of total phenolics and tannins were analyzed referring to the Folin-Ciocalteu reagent method (Pérez et al., 2023). Anthocyanin content was determined using the pH differential method (Lee et al., 2005), and total acidity was measured by alkali titration. Volatile aroma compounds were detected using gas chromatography-mass spectrometry (Agilent 7890B, GC-MS). Microbial carbon content was determined using the o-phenanthroline colorimetric method. The concentrations of calcium, potassium, phosphorus, iron, and other elemental ions were measured using inductively coupled plasma mass spectrometry (ICP-MS, Agilent 7900), and amino acid content was analyzed using liquid chromatography-mass spectrometry (LC-MS, Thermo Scientific).

### 2.10 Statistical analysis

For agronomic traits of pot-grown and field-grown plants, 10 replicates were used. All other measurements were performed with three independent biological replicates. Prior to parametric testing, data normality was assessed using the Shapiro–Wilk test (P >0.05 indicating normal distribution), and homogeneity of variances was evaluated using Levene’s test (P>0.05 indicating equal variances). When both assumptions were met, one-sided Student’s t-test and one-way ANOVA was used, followed by Tukey’s HSD *post-hoc* test for multiple comparisons (P<0.05). For data violating assumptions, the non-parametric Kruskal-Wallis rank-sum test was used, followed by Dunn’s *post-hoc* test with Bonferroni correction. For microbiome data, LEfSe was performed using the Kruskal-Wallis rank-sum test with pairwise Wilcoxon tests, and FDR correction (Benjamini-Hochberg, q<0.05) was applied. For amino acid data, one-way ANOVA with Tukey’s HSD was used, followed by FDR correction (Benjamini-Hochberg, q <0.05) across the 23 detected amino acids. For soil physicochemical properties and growth parameters, no additional multiple comparison correction was applied beyond Tukey’s HSD. All statistical analyses were performed using SPSS Statistics (version 26.0) and R software (version 4.1.0).

## 3 Results

### 3.1 Characteristics of GO

**Figure 1:**
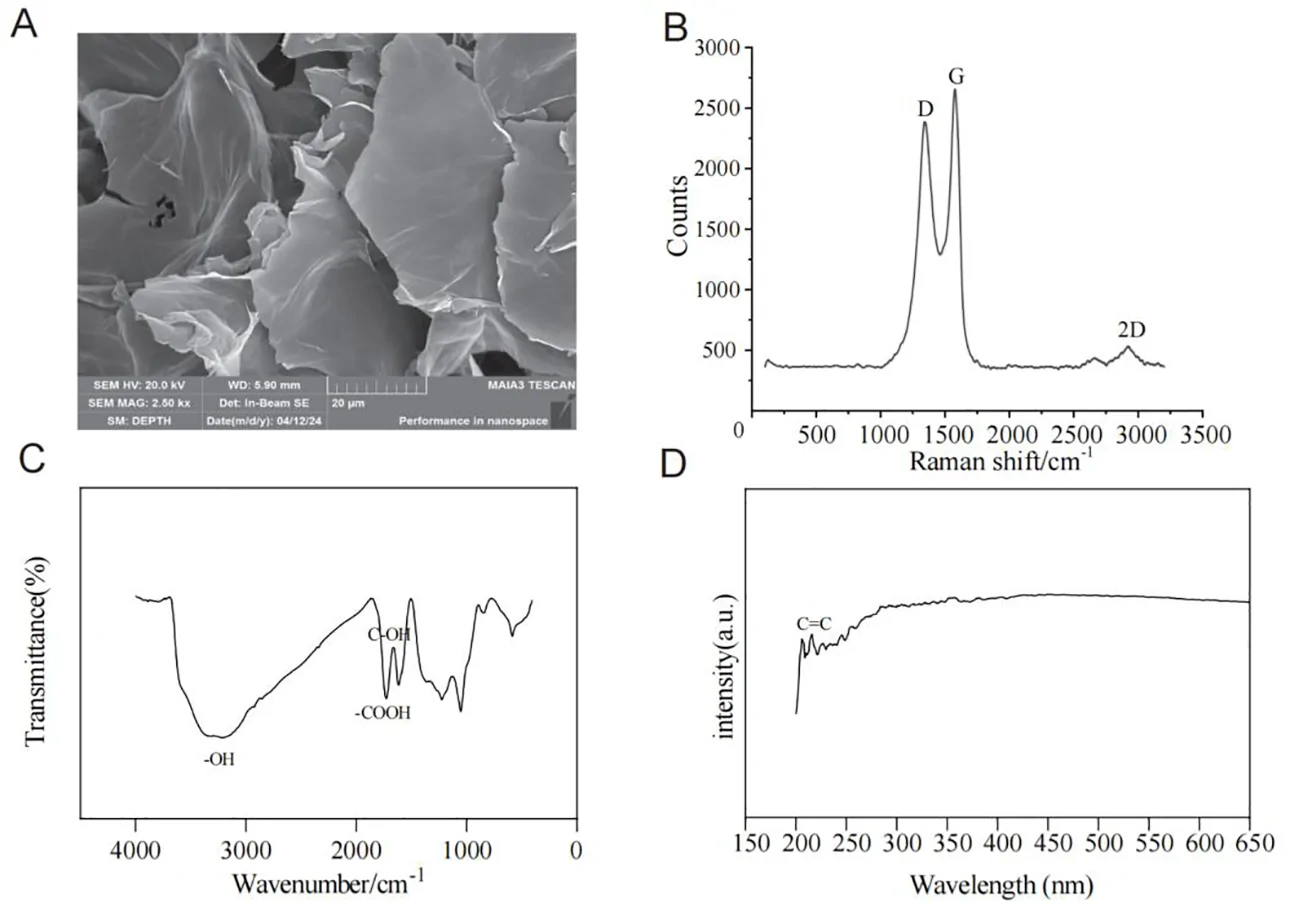
Characterization of GO. **(A)** SEM image; **(B)** Raman spectrum;**(C)** Infrared spectroscopy; **(D)** UV-Vis spectrum.

Scanning electron microscopy (SEM) showed that the graphene sample exhibited a stacked, folded, and distinctly layered morphology (**Figure 1A**). Its characteristic D and G bands were confirmed by Raman spectroscopy (**Figure 1B**). Furthermore, infrared spectroscopy identified surface oxygen-containing functional groups, such as C-OH (1630 cm^-^¹), C=O (1725 cm^-^¹), and -OH (3434 cm^-^¹) (**Figure 1C**). The UV-Vis spectrum of GO exhibited a major absorption peak at 230 nm, which is attributed to the π→π* transition within the C=C bonds (**Figure 1D**).

### 3.2 GO promoted the growth and development of grape seedlings

**Figure 2:**
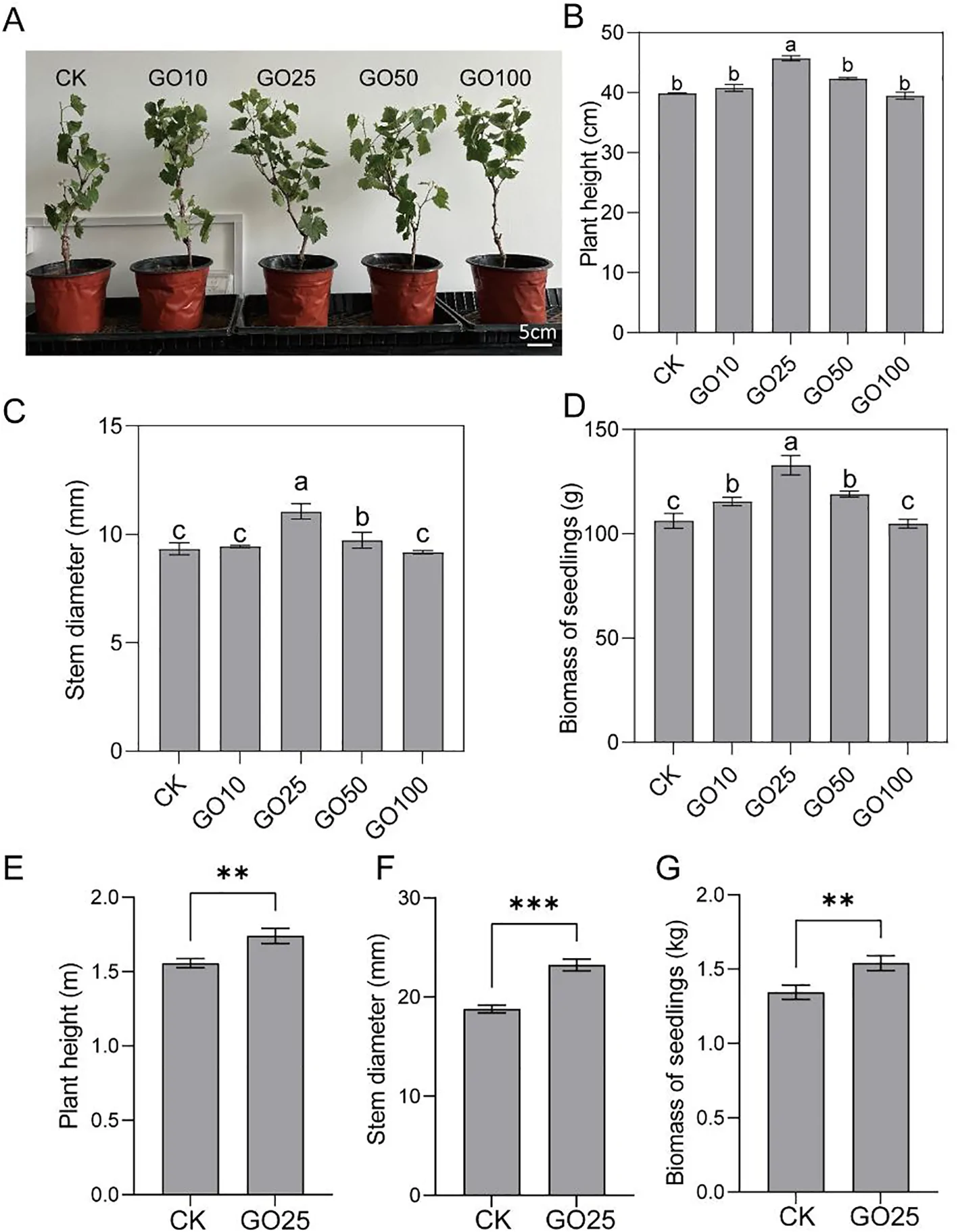
GO promotes the growth of grape seedlings. **(A)** Phenotypic photographs of grape seedlings treated with graphene; **(B)** Bar graph showing the plant height of grape seedlings treated with graphene; **(C)** Bar graph showing the stem diameter of grape seedlings treated with graphene; **(D)** Bar graph showing the biomass of grape seedlings treated with graphene. Data were analyzed by one-way analysis of variance (ANOVA), followed by Tukey’s HSD *post-hoc* test for multiple comparisons. Error bars show the standard errors. Different letters indicate significantly different groups (P < 0.05, n=10). The experiments were repeated three times with similar results. **(E–G)** Bar graphs showing the plant height, stem diameter, and biomass of grapevines in the field. Error bars from **(E-G)** indicate the standard errors (one-sided Student’s t-test, **P<0.01; ***P<0.001, n=10). The experiments were repeated three times with similar results.

After treating grape seedlings with different concentrations of graphene, the results showed that, compared with the control, 25 mg/L graphene significantly promoted the plant height of grape seedlings, with an increase of 14.6%, while the other concentrations had no significant effect on plant height. Regarding stem diameter, 25 mg/L and 50 mg/L graphene increased it by 18.5% and 39.3%, respectively, whereas 10 mg/L and 100 mg/L treatments showed no significant effect. In terms of biomass, 10, 25, and 50 mg/L graphene all exhibited significant promoting effects, with increases of 8.7%, 25.0%, and 12.0%, respectively, while the 100 mg/L treatment had no significant effect on biomass. Taken together, 25 mg/L graphene significantly promotes plant height, stem diameter, and biomass of grape seedlings, and can thus be recommended as the optimal concentration for field trials. Furthermore, under field conditions, treatment with 25 mg/L graphene significantly enhanced the growth of grape seedlings, resulting in increases of 11.8% in plant height, 23.6% in stem diameter, and 14.7% in biomass compared to the control group (**Figures 2E-G**). These results fully demonstrate the favorable application potential of graphene for promoting plant growth in agriculture.

### 3.3 GO enhances photosynthesis in grape seedlings

**Figure 3:**
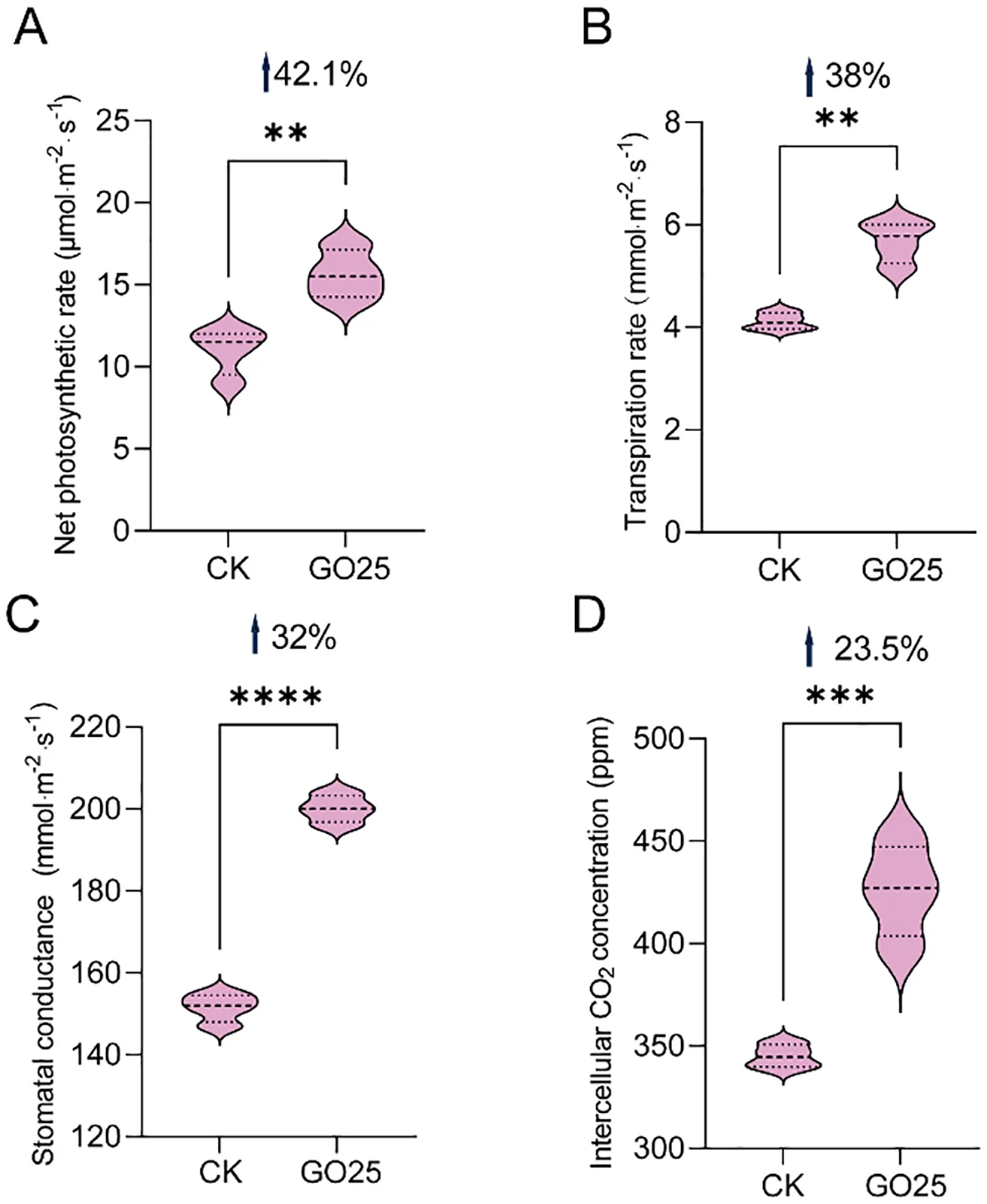
Effects of GO on photosynthesis in grape leaves. **(A)** Net photosynthetic rate; **(B)** Transpiration rate; **(C)** Stomatal conductance; **(D)** Intercellular CO_2_ concentration. Error bars from **(A-D)** indicate the standard errors (one-sided Student’s t-test, **P<0.01; ***P<0.001, ****P<0.0001, n=3). The experiments were repeated three times with similar results.

Field measurements of photosynthesis in grape leaves revealed that treatment with GO significantly enhanced the photosynthetic rate compared to the control. Specifically, the GO treatment increased the intercellular CO_2_ concentration, stomatal conductance, net photosynthetic rate, and transpiration rate by 23.5%, 32%, 42.1%, and 38%, respectively, relative to the control group (**Figure 3**).

### 3.4 GO reshapes the microbial community structure in the grape rhizosphere

**Figure 4:**
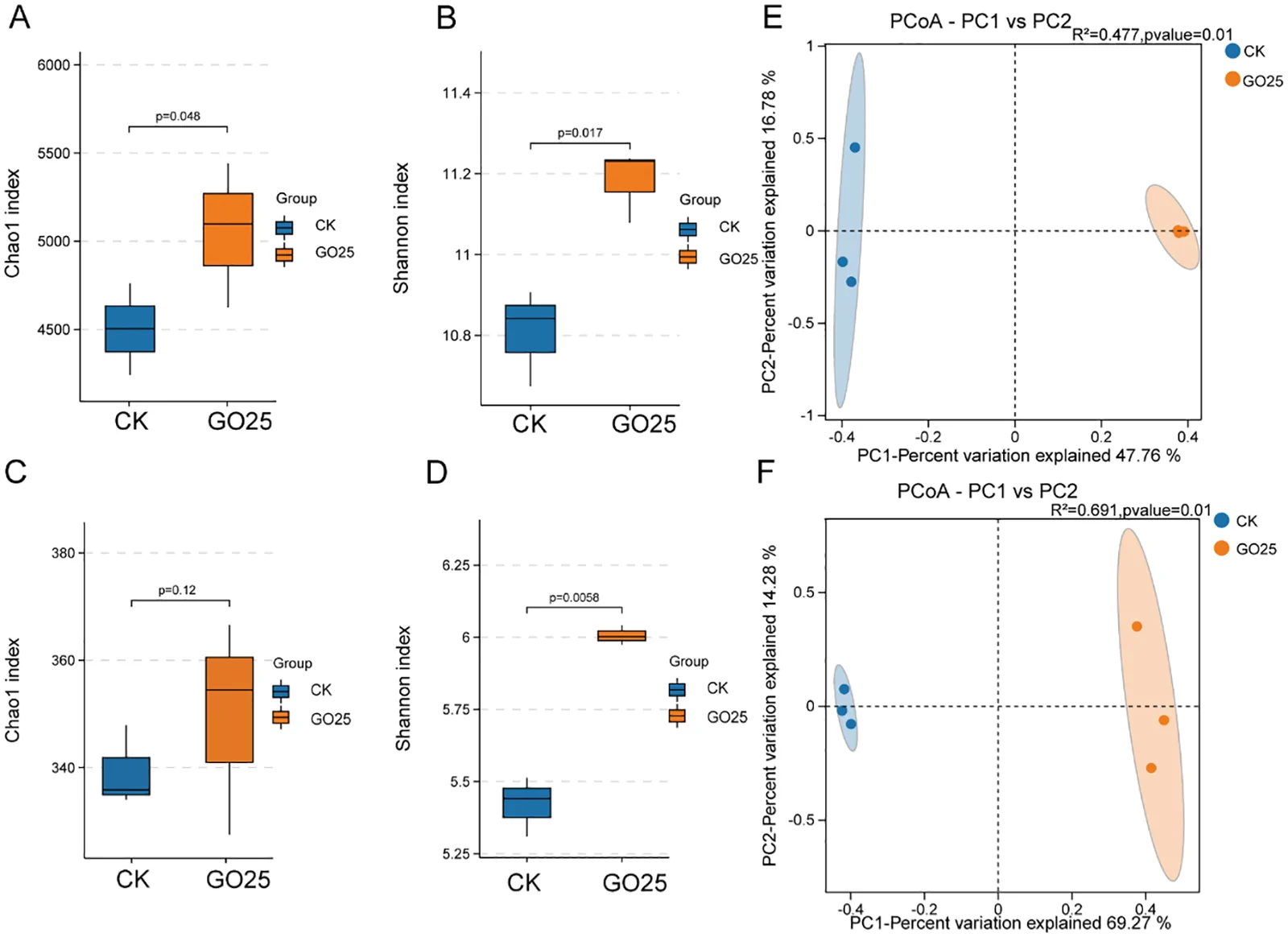
GO modifies the composition of the microbial community in the grape rhizosphere. **(A)** Bacterial Chao1 index in the grape rhizosphere soil samples with H_2_O or GO treatment at the OTU level; **(B)** Bacterial Shannon index in the grape rhizosphere soil samples with H_2_O or GO treatment at the OTU level; **(C)** Fungal Chao1 index in the grape rhizosphere soil samples with H_2_O or GO treatment at the OTU level; **(D)** Fungal Shannon index in the grape rhizosphere soil samples with H_2_O or GO treatment at the OTU level. For A-D, the significant differences between groups were tested via the Wilcoxon test, n=3. p<0.05 indicates statistically significant differences between sample treatments. **(E)** PCoA analysis of rhizosphere bacterial β-diversity of grape plants cultivated in soil; **(F)** PCoA analysis of rhizosphere fungal β-diversity of grape plants cultivated in the grape rhizosphere soil. For E, F, bray–curtis dissimilarity tests were performed on the taxonomic profile (at the OTU level, n=3) for CK and GO microbial.

The collected samples were subjected to sequencing, with all samples achieving coverage rates above 95%. The rarefaction curve analysis indicated that the sequencing depth was adequate for downstream analysis (**Supplementary Figures 1A, B**). A total of 392130 16S rRNA raw reads and 480433 ITS raw reads were generated from the soil sample DNA. After quality screening of all sequencing sequences, 356325 16S rRNA effective tags and 407472 ITS effective tags were obtained. Using a 97% similarity threshold, effective bacterial tags were clustered into operational taxonomic units (OTUs) for grape rhizosphere soil. To compare the bacterial and fungal communities in the soybean rhizosphere, Venn diagrams were constructed based on OTU profiles. The analysis revealed a distinct separation between the control (CK) and graphene oxide-treated (GO) groups. Specifically, 11,974 and 13,474 bacterial OTUs were unique to CK and GO, respectively, with only 1,149 shared. Similarly, 645 and 713 fungal OTUs were unique to CK and GO, with 81 shared. The significantly higher number of unique OTUs in the GO treatment for both kingdoms suggests that GO amendment markedly increased the richness of rhizosphere microbial communities compared to the control (**Supplementary Figures 1C, D**). Furthermore, α-diversity analysis at the OTU level shows that GO significantly enhances bacterial diversity, as indicated by higher Chao1 and Shannon indices, as well as fungal diversity (Chao1 index) (**Figures 4A, B, D**), but has no significant effect on the fungal Chao index, compare to the control (**Figure 4C**). Principal Coordinate Analysis (PCoA) was conducted to assess β-diversity, revealing significant differences in bacterial and fungal communities between CK and GO (Bray-Curtis, PERMANOVA, p<0.05) (**Figures 4E**, **F**). These results suggest that GO promotes both richness and diversity in rhizosphere bacterial and fungal communities relative to the control.

**Figure 5:**
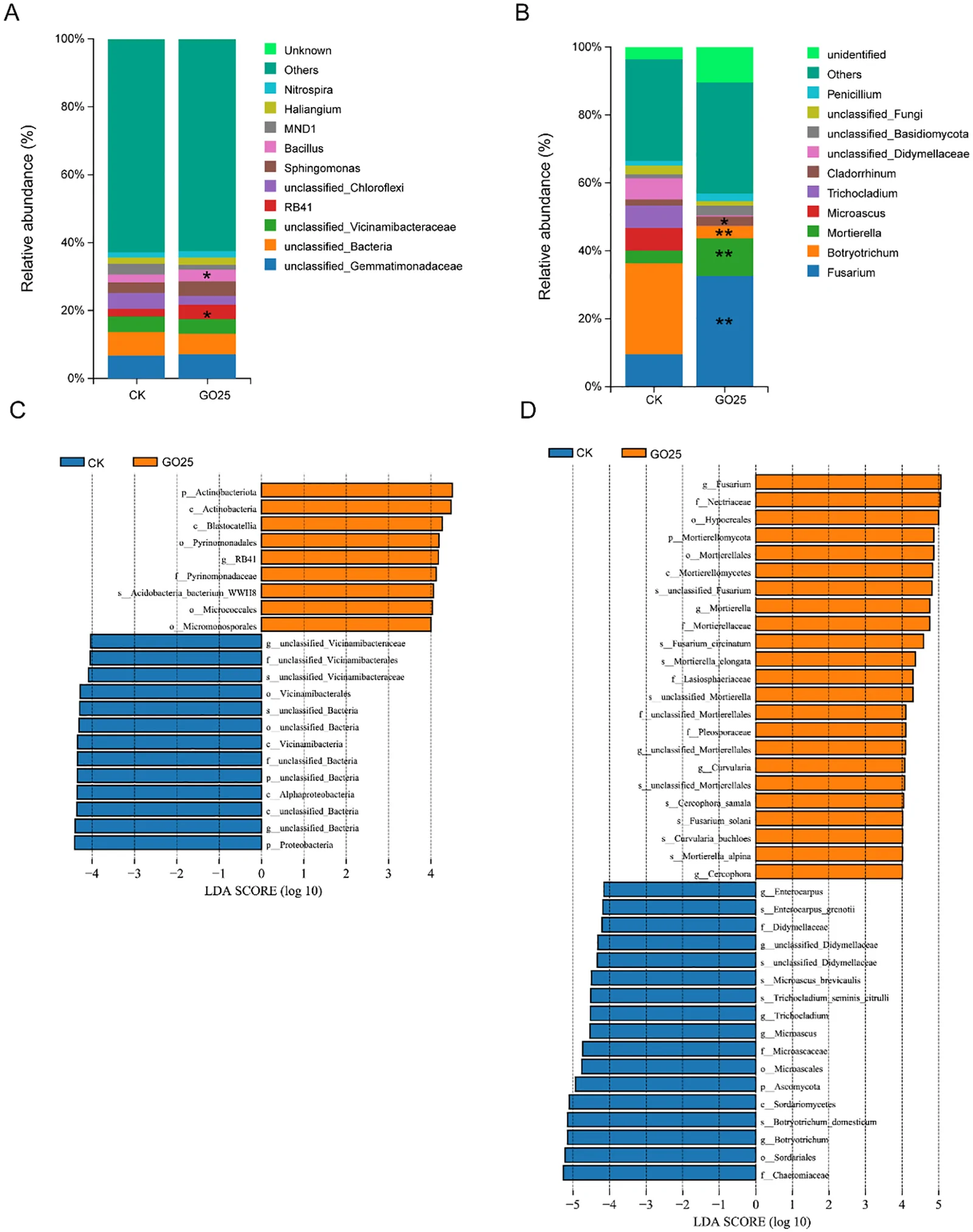
Analysis of the microbial community composition in grape rhizosphere soil following GO treatment. **(A)** Bar plot (stacked) showing Top 10 of bacterial community composition at the genus level; **(B)** Bar plot (stacked) showing Top 10 of fungal community composition at the genus level; **(C)** LEfSe linear discriminant analysis for bacteria; **(D)** LEfSe linear discriminant analysis for fungi.

The analysis of bacterial species composition revealed that the top10 genera were *unclassified_Gemmatimonadaceae*, *unclassified_Bacteria*, *unclassified_Vicinamibacteraceae*, *RB41*, *unclassified_Chloroflexi*, *Sphingomonas*, *Bacillus*, *MND1*, *Haliangium*, and *Nitrospira*. At the genus level, the top 10 fungal taxa were *Fusarium*, *Botryotrichum*, *Mortierella*, *Microascus*, *Trichocladium*, *Cladorrhinum*, *unclassified_Didymellaceae*, *unclassified_Basidiomycota*, *unclassified_Fungi*, and *Penicillium*. Further comparative analysis revealed that, compared to the control, treatment with nanosol humic acid promoted the abundance of plant-beneficial microbial groups such as *RB41*, *Sphingomonas*, *Bacillus*, and *Mortierella*, while suppressing harmful microorganisms including *Fusarium oxysporum* and *Trichothecium* (**Figure 5**). This shift contributes to the formation of rhizosphere immunity, thereby supporting plant and soil health.

The Kruskal-Wallis rank-sum test was employed to compare differences among treatment groups, with FDR correction (Benjamini-Hochberg, q < 0.05). All analyses were based on three independent biological replicates per group (n = 3).

### 3.5 GO increased the nutrient content in grape rhizosphere soil

**Figure 6:**
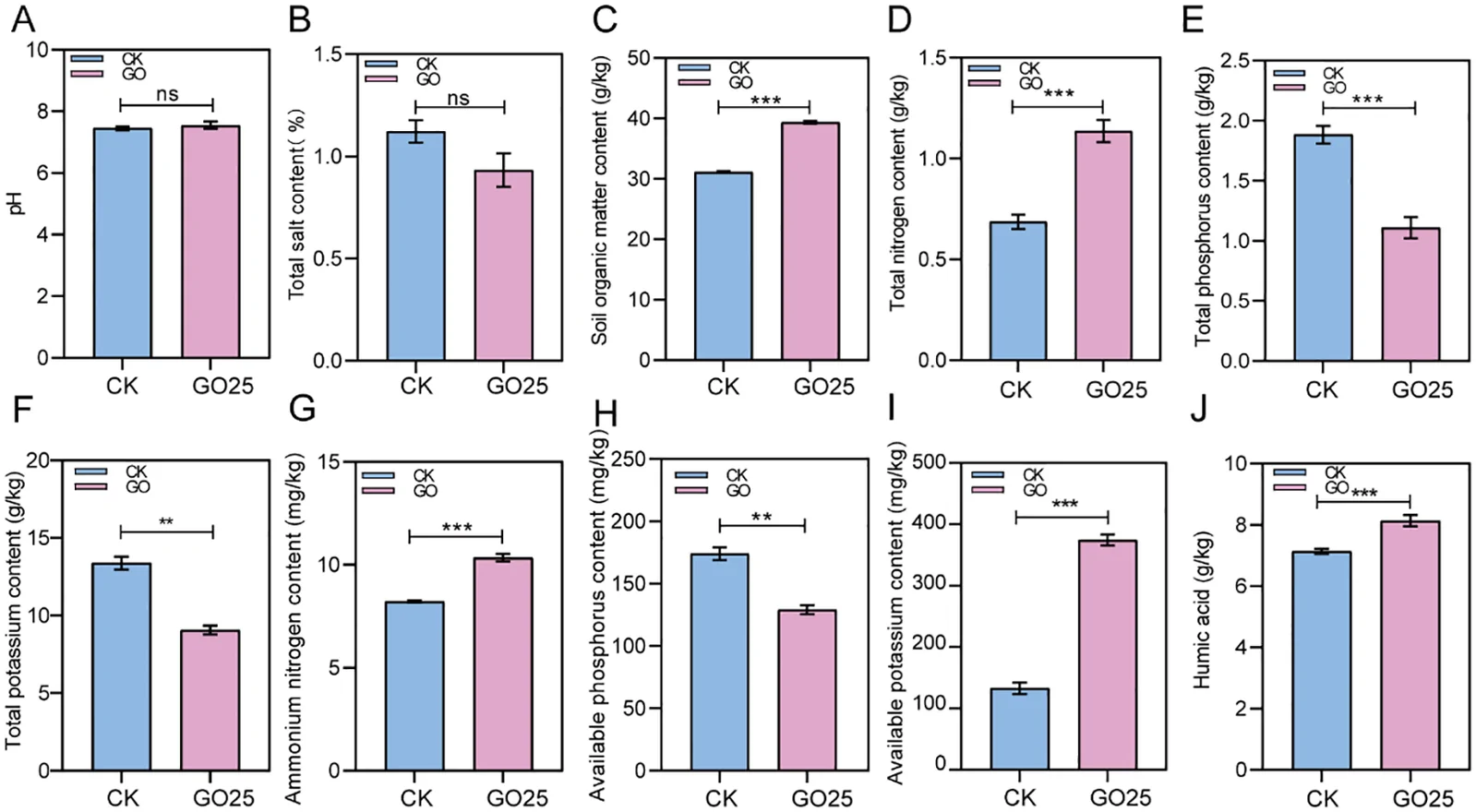
Physicochemical properties of rhizosphere soil of grape. **(A)** pH; **(B)** Total salt content; **(C)** SOM; **(D)** TN; **(E)** TP; **(F)** TK; **(G)** NH4^+^-N; **(H)** AP; **(I)** AK; **(J)** HA. Asterisks indicate significant differences (one-sided Student’s t-test, **p<0.01, ***p<0.001, ns indicates non-significant differences, p≥0.05, n=3).

Physicochemical analysis of the rhizosphere soil of ‘Crimson Seedless’ grape showed that, compared with the control, GO significantly increased the contents of soil organic matter (SOM), total nitrogen (TN), ammonium nitrogen (NH_4_^+^-N), available potassium (AK), and humic acid (HA), with enhancement ratios of 26.29%, 65.57%, 26.02%, 181.79%, and 14.05%, respectively. It inhibited the contents of total phosphorus (TP), total potassium (TK), and available phosphorus (AP), while showing no significant effects on soil pH or total salt content (TS) (**Figure 6**).

### 3.6 GO enhances grape berry quality

To further clarify the impact of GO on grape berry quality, we collected grape samples and analyzed their external quality and internal nutritional components. The results showed that berries treated with GO exhibited superior performance in longitudinal diameter, single-berry weight, fruit shape index, fruit firmness, and bunch weight compared to both the untreated control (CK) and the BR treatment (BR refers to a commercially available fertilizer). The order of effectiveness was GO > BR > CK. Specifically, compared to the control, GO treatment increased these parameters by 14.9%, 5.3%, 15.5%, 18.9%, and 7.32%, respectively. Additionally, GO treatment resulted in a reduction in transverse diameter compared to the control (**Table 1**). These findings indicate that GO improves the external quality of grapes.

**Table 1:** Statistical analysis of grape berry appearance quality under different treatments.

Treatment	Transverse diameter (cm)	Longitudinal diameter (cm)	Berry weight (g)	Berry shape index	Fruit firmness (Postharvest 6d, kg·cm^-2^)	Cluster weight (g)
CK	2.51 ± 0.01a	3.23 ± 0.23b	8.23 ± 0.02c	1.29 ± 0.10b	2.81 ± 0.06b	951.81 ± 8.19b
GO25	2.49 ± 0.06a	3.71 ± 0.06a	8.69 ± 0.14a	1.49 ± 0.06a	3.34 ± 0.04a	1021.46 ± 21.76a
BR	2.53 ± 0.05a	3.35 ± 0.04b	8.50 ± 0.59b	1.32 ± 0.02b	2.73 ± 0.03b	973.22 ± 8.14b

Data are presented as mean ± SD, CK refers to the control group; GO25 refers to the treatment with 25 mg/L of GO; BR refers to Bayer fertilizer. Data were analyzed by one-way ANOVA, Tukey’s HSD test, n=3,with different letters indicating significant differences.

To further elucidate the effect of GO on grape edible quality, we examined changes in nutrient content. The results revealed that both GO and BR treatments enhanced the contents of total sugars, Vitamin C content, soluble solids, tannins, total anthocyanins, calcium, and iron compared to the control, with GO demonstrating more pronounced promotion. The increases achieved by GO reached 18.1%, 7.6%, 14.8%, 9.5%, 25%, and 35.3%, respectively. Furthermore, GO treatment suppressed the contents of total acidity, potassium, and phosphorus. No significant effect was observed on total phenolic content (**Table 2**). Furthermore, we detected the contents of 24 amino acids in grape berries. Comparative analysis revealed that the amino acid content was highest under GO treatment, followed by BR treatment, and lowest in the CK control. Compared with CK, GO treatment significantly promoted the accumulation of the following amino acids: 1-Methylhistidine, 3-Methylhistidine, 4-Aminobutyric acid, 4-Hydroxyproline, Glycine, L-Alanine, L-Arginine, L-Asparagine, L-Aspartic acid, L-Citrulline, L-Glutamic acid, L-Glutamine, L-Histidine, L-Lysine, L-Methionine, L-Ornithine, L-Phenylalanine, L-Proline, L-Threonine, L-Tryptophan, L-Tyrosine, L-Valine, and Serine(**Supplementary Table 1**).

**Table 2:** Statistical analysis of grape berry nutritional quality components under different treatments.

Treatment	Total sugar content (mg/g)	Total acid content (mg/mL)	Vitamin C content (mg/kg)	Soluble solid content (%)	Total Phenols content (%)	Tannin content (mg/kg)	Anthocyanin content (mg/g)	Calcium content (mg/g)	Potassium content (mg/g)	Phosphorus content (mg/g)	Iron content (mg/g)
CK	103.88±8.71^b^	5.38±0.70^a^	105.92±6.49	13.50±1.04^b^	0.02±0.00^a^	321.46±3.54^b^	0.12±0.05^c^	0.11±0.03^b^	1.31±0.21^a^	0.24±0.03^a^	5.63±0.92^b^
GO25	122.73±0.55^a^	5.15±0.27^a^	113.92±2.95	15.50±0.71^a^	0.02±0.01^a^	352.03±14.97^a^	0.15±0.01^a^	0.15±0.01^a^	1.25±0.07^a^	0.18±0.02^b^	7.62±0.82^a^
BR	115.43±5.10^ab^	4.98±1.20^a^	108.37±2.47	15.80±0.71^a^	0.02±0.01^a^	350.07±2.66^a^	0.14±0.01^b^	0.14±0.01^ab^	1.43±0.10^a^	0.26±0.01^a^	7.27±0.74^a^

Data are presented as mean ± SD, CK refers to the control group; GO25 refers to the treatment with 25 mg/L of GO; BR refers to Bayer fertilizer. Data were analyzed by one-way ANOVA, Tukey’s HSD test, n=3, with different letters indicating significant differences.

### 3.7 Redundancy analysis of microbial community composition and physicochemical properties

**Figure 7:**
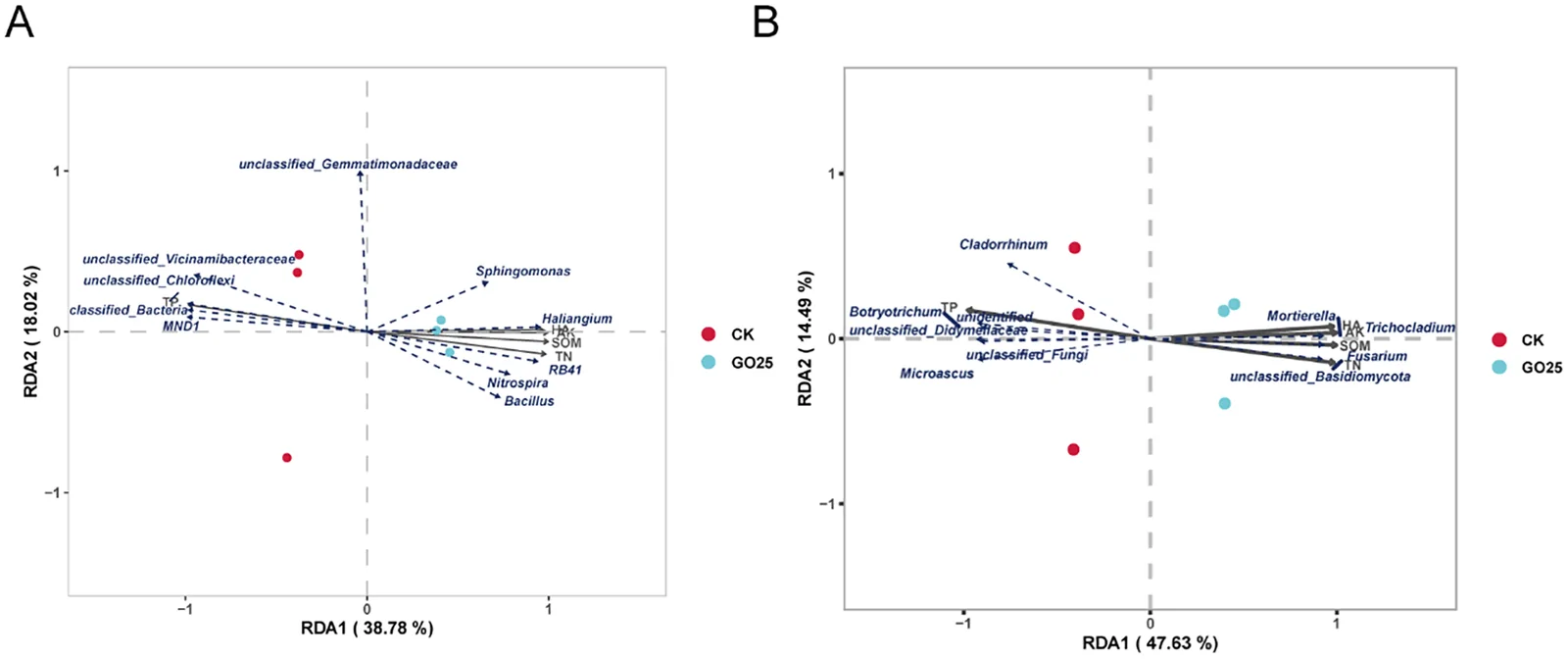
Distance-based RDA of grape rhizosphere microbial communities and soil physicochemical properties. **(A)** RDA of bacterial communities and soil physicochemical properties; **(B)** RDA of fungal communities and soil physicochemical properties. Arrows represent environmental factors and species, with filled circles denoting sample groups. An acute angle between arrows indicates a positive correlation, whereas an obtuse angle signifies a negative correlation.

To further investigate the correlation between changes in soil physicochemical properties and microbial communities in the grape rhizosphere, RDA was performed based on genus-level bacterial community diversity and physicochemical properties. The results showed that the first and second axes explained 38.78% and 18.02% of the total variation in bacterial community composition and physicochemical properties in the grape rhizosphere soil, respectively. Specifically, *Sphingomonas*, *Haliangium*, *RB41*, *Bacillus*, and *Nitrospira* were positively correlated with SOM, HA, AK, and TN, but negatively correlated with TP. In contrast, *unclassified_Gemmatimonadaceae*, *unclassified_Vicinamibacteraceae*, *unclassified_Chloroflex*, *unclassified_Bacteria*, and *MND1* were positively correlated with TP and negatively correlated with SOM, HA, AK, and TN (**Figure 7A**). Additionally, redundancy analysis based on genus-level fungal community diversity and physicochemical properties revealed that the first and second axes explained 47.63% and 14.49% of the total variation in fungal community composition and physicochemical properties in the grape rhizosphere soil, respectively. Notably, *Mortierella*, *Trichocladium*, *Fusarium*, and *unclassified_Basidiomycota* were positively correlated with SOM, HA, AK, and TN, but negatively correlated with TP. Conversely, *Cladorrhinum*, *Botryotrichum*, *Microascus*, *unclassified_Didymellaceae*, and *unclassified_Fungi* were positively correlated with TP and negatively correlated with SOM, HA, AK, and TN (**Figure 7B**).

## 4 Discussion

GO exhibits a light, gauze-like, transparent flake structure with slight wrinkles and undulations on the surface. The sheet thickness is approximately 3 nm, consisting of about 5 layers, and it is primarily composed of the two elements, carbon (C) and oxygen (O) (**Figure 1**). Owing to its high specific surface area and abundant oxygen-containing functional groups, exhibits broad application prospects in agriculture. Numerous studies have shown that graphene can promote plant growth (Guo et al., 2021; Chen et al., 2022; Cao et al., 2024; Qiao et al., 2025a); however, its role in regulating fruit quality and the underlying mechanisms remain unclear. To address this, the present study introduced exogenous graphene to observe changes in grapevine growth, analyze the succession of rhizosphere soil microbial community structure and soil physicochemical properties, and further evaluate the responsive characteristics of grape fruit quality, aiming to elucidate the interaction mechanism between graphene and grapevines.

### 4.1 GO promotes plant growth and development

The regulation of plant growth by graphene occurs at multiple levels. In this study, graphene significantly promoted the growth of grape seedlings (**Figures 2**, **3**). GO can enhance root growth and development. Graphene tends to adsorb onto the metabolically active root tip meristem and elongation zones, and its enrichment effect increases significantly with higher treatment concentrations (Chen et al., 2022). Graphene can enter plant cells via endocytosis and interact with intracellular biomolecules (Dąbrowski et al., 2023). Further molecular-level studies have shown that graphene affects the expression levels of enzymes involved in key respiratory pathways in plant roots, including glycolysis, pyruvate metabolism, and the tricarboxylic acid cycle, thereby enhancing root respiration and promoting the production of ATP, which in turn stimulates root growth (Chen et al., 2022). On one hand, graphene nanomaterials improve the rhizosphere microenvironment and enhance the efficiency of water and nutrient uptake, thus promoting root growth. Graphene also increases the root uptake efficiency of mineral elements such as nitrogen, phosphorus, and potassium. The high surface area and strong adsorption capacity of graphene enhance the availability of rhizosphere nutrients while improving soil pore structure, facilitating nutrient absorption by roots (Chen et al., 2021). When combined with trace elements, graphene nanomaterials can form stable complexes, thereby improving the delivery efficiency of these nutrients (Yin et al., 2018). In this study, after exogenous application of graphene under both greenhouse and field conditions, we observed that, compared with the control, 25 mg/L and 50 mg/L graphene significantly promoted grape seedling growth in terms of plant height and stem diameter, as well as enhancing leaf photosynthesis. This finding is consistent with most previous studies and indicates that graphene has an excellent growth-promoting function. However, the effects of graphene on different plant species vary depending on the cultivar, and for many plants, there is no concentration-dependent response. In this study, we observed that a high concentration of GO (100 mg/L) had no significant effect on plant growth (**Figure 2**). This may be attributed to the fact that high concentrations of graphene can disrupt the plant’s antioxidant enzyme system and inhibit the absorption of key nutrient ions (Liang et al., 2025).

### 4.2 GO ameliorates plant rhizosphere microecology

Soil microorganisms are essential for maintaining soil health and promoting plant growth. In this study, a rhizosphere addition method of graphene was employed. The results showed that, compared with the control, graphene significantly altered the rhizosphere microbial community structure, increased the abundance and diversity of the microbial community (**Figure 4**), and notably enriched potentially beneficial microbial taxa in the rhizosphere, including *Bacillus*, *Sphingomonas*, *Trichoderma*, and *Mortierella* (**Figure 5**). Among these, *Bacillus*, *Sphingomonas*, and *Trichoderma* have been reported to promote plant growth (Sun et al., 2022; Gatheru et al., 2017; Chen et al., 2024), while *Mortierella* and *RB41* have been shown to participate in soil carbon cycling and enhance soil nutrient availability (Stone et al., 2021). However, the ecological functions of these differentially abundant microbial taxa, identified at the genus level, require further validation. This selective enrichment phenomenon may be attributed to the specific adsorption of microorganisms induced by specialized substances derived from graphene (Cao et al., 2024). Previous studies have demonstrated that graphene can promote soybean growth and yield by increasing the abundance of rhizobia in the soybean rhizosphere (Qiao et al., 2025a).

GO is a novel two-dimensional atomic crystal material consisting of a single layer of carbon atoms connected by sp² hybridization, possessing excellent properties such as high electrical conductivity and large specific surface area. Studies have shown that upon entering the soil environment, graphene exerts significant effects on plants and microorganisms, with its impact exhibiting a clear concentration-dependent pattern, manifesting as either stimulatory or inhibitory effects (Qiao et al., 2025b; Ahmed and Rodrigues, 2013; Al-Jumaili et al., 2017). In natural ecosystems, plant litter serves as a major carbon input, and its decomposition is primarily regulated by soil physicochemical properties and microbial community activities. The alterations in soil physicochemical properties, microbial activity, and community structure induced by graphene introduction may substantially affect litter decomposition processes. The results of this study demonstrated that exogenous application of graphene significantly increased the contents of soil organic matter (SOM), total nitrogen (TN), ammonium nitrogen (NH_4_^+^-N), available potassium (AK), and humic acid (HA), with enhancement rates of 26.29%, 65.57%, 26.02%, 181.79%, and 14.05%, respectively. Conversely, it inhibited the contents of total phosphorus (TP), total potassium (TK), and available phosphorus (AP), while exhibiting no significant effect on soil pH or total salt (TS) content (**Figure 6**), indicating efficient uptake of soil phosphorus and potassium by plants. RDA was conducted to elucidate the correlations between grapevine rhizosphere microorganisms and soil nutrients. The results showed that *Mortierella*, *Trichocladium*, *Fusarium*, and *unclassified_Basidiomycota* were positively correlated with SOM, HA, AK, and TN, but negatively correlated with TP. In contrast, *Cladorrhinum*, *Botryotrichum*, *Microascus*, *unclassified_Didymellaceae*, and *unclassified_Fungi* exhibited significant positive correlations with TP, and significant negative correlations with SOM, HA, AK, and TN (**Figure 7**). These findings indicate that graphene improves soil physicochemical properties and enhances soil fertility by inducing the enrichment of functional microorganisms, a phenomenon consistently observed in studies on graphene-mediated amelioration of leguminous plant soils (Chen et al., 2024; Qiao et al., 2025a).

### 4.3 Environmental behavior and toxicity of GO

It is noteworthy that GO can exert harmful effects on certain biological cells through mechanisms including extracellular encapsulation, intracellular oxidative stress, or direct disruption of cell membranes. Moreover, even at low concentrations where no apparent toxicity is observed, GO can form complexes with other substances, thereby inducing toxic effects on organisms. These findings indicate that GO poses potential adverse effects on the ecological environment and exhibits biotoxicity to certain microorganisms (Ge et al., 2016; Selck et al., 2016). In a study on the transport of few-layer GO using ¹^4^C-labeled graphene oxide, Lu et al. (2017) demonstrated that GO could be taken up by zebrafish and retained within the body, with large FLG primarily accumulating in the intestine, whereas small FLG were distributed in both the intestine and the liver. Furthermore, the inhibitory effects of graphene oxide on microorganisms are attributed to three main mechanisms: physical puncturing of cell membranes by sharp edges, oxidative stress caused by reactive oxygen species generation, and physical encapsulation that limits nutrient acquisition and respiration (Ahmed and Rodrigues, 2013; Al-Jumaili et al., 2017). However, other studies have also shown that GO at appropriate concentrations does not pose biosafety concerns. For instance, previous studies have demonstrated the existence of microorganisms capable of utilizing and degrading graphene as a sole carbon source in the environment (Qu et al., 2018). The research group led by Professor Liang Mao at the School of the Environment, Nanjing University, employed ¹^4^C-labeled graphene to investigate its accumulation, distribution, translocation, and transformation in rice. The results revealed that graphene could penetrate the cell wall and cell membrane of rice leaves, entering the chloroplasts, and hydroxyl radicals were detected in the leaves that had taken up graphene. The substantial amount of ¹^4^CO_2_ captured in the experiments confirmed that graphene taken up by rice shoots and leaves could be mineralized (Huang et al., 2018). Furthermore, the combination of graphene with exogenous microbial inoculants accelerated litter decomposition in soil (Song et al., 2024). White-rot fungi, which are widespread in nature, can accelerate graphene degradation by secreting lignin peroxidases (Lalwani et al., 2014). Based on this, it can be inferred that graphene introduction into soil may promote an increase in the abundance of related microbial communities. Additionally, graphene shares structural similarities with polycyclic aromatic hydrocarbons (PAHs) and lignin. While previous studies have extensively reported the use of white-rot fungi for PAH degradation, recent research has also found that appropriate amounts of graphene can increase the population of PAH-degrading microorganisms, thereby significantly influencing the environmental behavior of PAHs in soil (Mu et al., 2019).

Graphene nanomaterials exhibit a concentration-dependent regulatory effect on microbial metabolism. At an optimal low concentration (50 mg/L), graphene significantly increased microbial biomass (4.5-fold) and enhanced caproic acid production by 42.8%, acting as both a conductive scaffold for biofilm formation and an electron shuttle to improve interspecies electron transfer and intracellular ATP levels. In contrast, a high concentration (125 mg/L) disrupted microbial balance, inhibited biofilm formation, and redirected carbon flux toward low-value metabolites, thereby suppressing caproic acid synthesis. Transcriptomic analysis revealed that graphene upregulated genes involved in fatty acid synthesis, electron transfer, quorum sensing, and energy metabolism. Furthermore, graphene oxide (GO) can serve as a synergist for chemical pesticides; for example, GO as a nanocarrier significantly improved the thermal stability and UV tolerance of Beauveria bassiana conidia, enhancing fungal survival under field conditions (Zhan et al., 2026). Moreover, the combination of high-concentration GO with carbendazim disrupted glutathione on the cell membrane through electron transfer, thereby attenuating fungal cell activity and enhancing the antifungal activity of carbendazim (Hu et al., 2021).

### 4.4 Effects of graphene oxide on plant fruit quality

**Figure 8:**
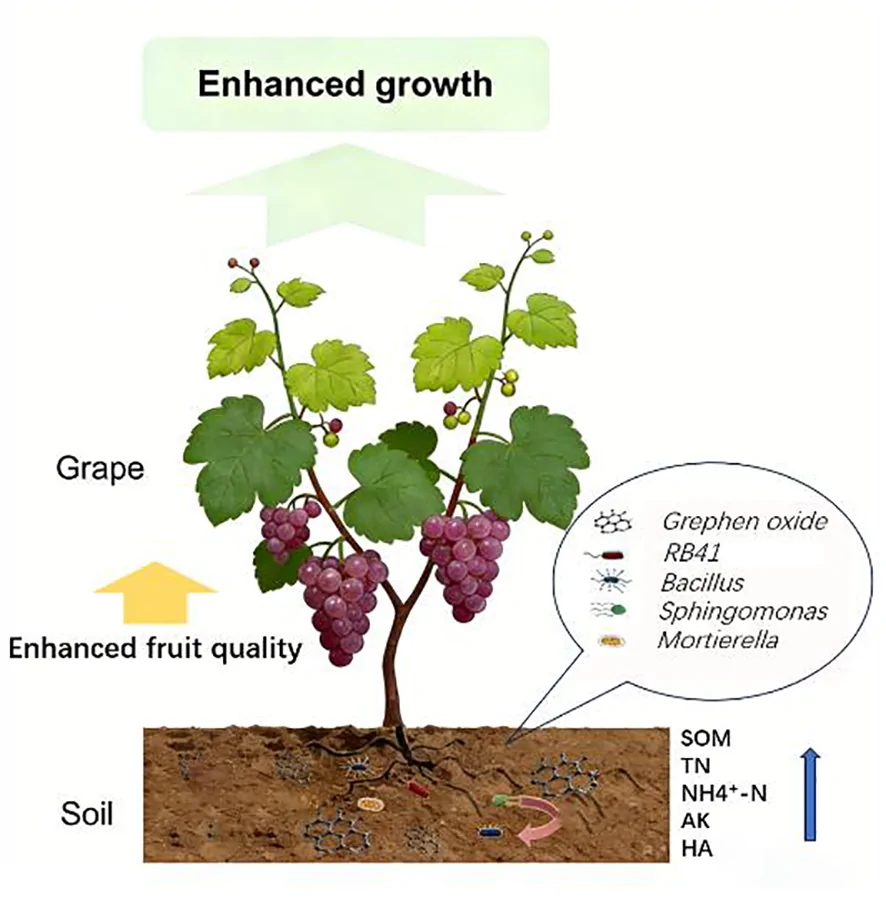
Proposed model for GO promoting grape growth. GO not only promotes grape growth but also improves fruit quality. These effects are achieved by modulating the diversity of the rhizosphere microbial community, enriching potentially beneficial microorganisms such as *Bacillus*, *Sphingomonas*, and *Mortierella*, along with improving soil fertility.

To date, research has demonstrated that graphene significantly enhances the growth and stress tolerance of various plant species. However, reports on its use as a fertilizer to regulate fruit quality remain very limited. Existing studies have mostly focused on the effects of graphene as a building material on fruit quality (Li et al., 2022). In this study, we examined the effect of graphene treatment on the external quality and internal nutritional flavor of grapes. Compared with the commercially available fertilizer BR and a control group not irrigated with graphene, graphene treatment significantly improved external quality traits, including berry weight, fruit shape index, and fruit firmness (**Table 1**). Furthermore, GO also enhanced the internal flavor of grapes, notably increasing the contents of total sugars, the sugar-to-acid ratio, vitamin C, soluble solids, tannins, and nutrients such as calcium. We further measured the contents of 24 amino acids; the results showed that, compared with the control, GO induced an increase in the levels of most amino acids, thereby improving the overall nutritional value of the grapes (**Table 2**; **Supplementary Table 1**). This finding is highly novel, as current reports on graphene-mediated regulation of fruit quality are scarce. This study provides a theoretical foundation for the future application of graphene to regulate fruit quality in agriculture and forestry. Regarding the mechanism by which graphene improves grape quality, we propose that: on the one hand, graphene can enter plant leaves and localize in chloroplasts, where it increases chloroplast volume and the number of starch grains and mitochondria, thereby enhancing photosynthetic efficiency and promoting the synthesis of organic compounds (Younes et al., 2019); on the other hand, graphene may enhance fruit quality and yield by strengthening plant stress tolerance and metabolic capacity, as well as by modulating the expression of genes involved in root development (Jiao et al., 2025; Yan et al., 2024; Cao et al., 2024). In summary, GO can regulate plant life activities at multiple levels and dimensions, promoting both plant growth and fruit quality (**Figure 8**). This offers theoretical guidance for the use of nanomaterials to improve plant productivity and fruit quality within the context of sustainable agriculture. Nevertheless, the specific molecular mechanisms through which graphene influences the intrinsic quality of fruits require further investigation.

## 5 Conclusion

This study demonstrates that exogenous application of GO at an appropriate concentrations 25 mg/L can significantly promote the growth, biomass accumulation, and photosynthetic capacity of ‘Crimson Seedless’ grape seedlings, as evidenced in both pot experiments and field trials. Additionally, GO treatment improved the rhizosphere microecology, enriched potentially beneficial microorganisms such as *Bacillus*, *Sphingomonas*, *RB41*, and *Mortierella*, and increased the contents of soil organic matter, total nitrogen, ammonium nitrogen, available potassium, and humic acid, although it exerted certain inhibitory effects on phosphorus and potassium elements. Furthermore, GO treatment significantly enhanced fruit weight, firmness, and fruit shape index, as well as the levels of total sugar, vitamin C, soluble solids, tannins, anthocyanins, calcium, and 23 amino acids (**Figure 8**). Our results demonstrate that go application is associated with altered rhizosphere microbial communities and improved grape growth performance. In summary, GO exhibits positive potential in regulating grape growth and fruit quality, thereby providing both theoretical and experimental foundations for its agricultural application.

## Data Availability

The original contributions presented in the study are publicly available. This data can be found here: NCBI, PRJNA1508925.
