# Supplementary material for: Effects of graphene oxide on grape growth, rhizosphere microbial diversity, and berry quality
Source: Front Plant Sci. 2026 Aug 26;17:1941614. doi: 10.3389/fpls.2026.1941614 (PMC13555696; doi:10.3389/fpls.2026.1941614)
Supplement: Supplementary file 1 [file DataSheet1.pdf]

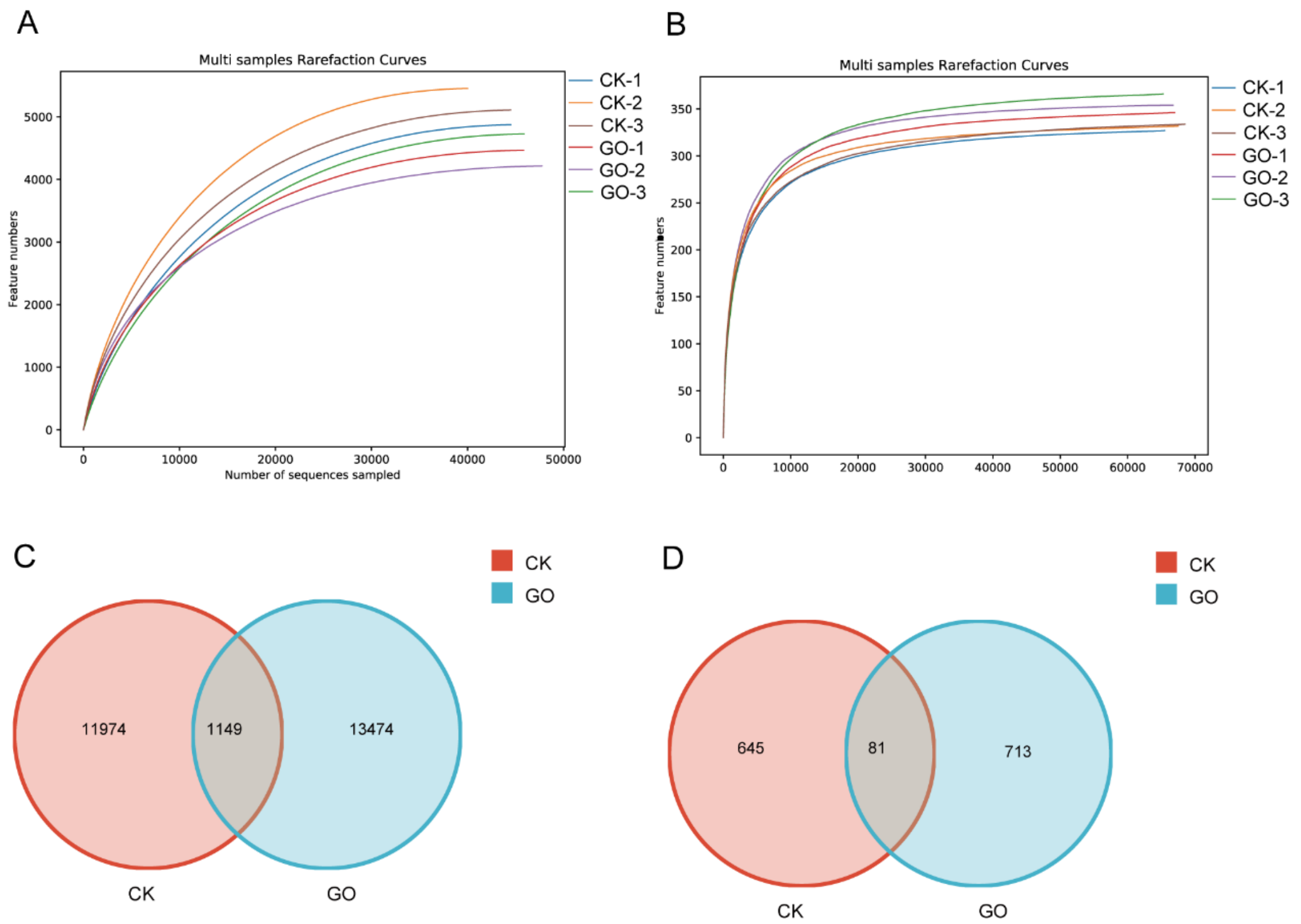

Figure S1 Analysis of the diversity of grape rhizosphere microbial communities

(A) Rarefaction curve of bacteria based on OTU levels; (B) Rarefaction curve of fungi based on OTU levels. Venn diagrams of (C) bacterial OTU quantity and (D) fungal OTU quantity, with three independent biological replicates per group.

Table S1 Statistical analysis of grape berry amino acid contents under different treatments

Treatment	1-Methyhistidine (nmol/g)	3-Methyhistidine (nmol/g)	4-Aminobutyric acid (nmol/g)	4-Hydroxyproline (nmol/g)	Beta-Alanine (nmol/g)	Glycine (nmol/g)	L-Alanine (nmol/g)	L-Arginine (nmol/g)	L-Asparagine (nmol/g)	L-Aspartic acid (nmol/g)	L-Citrulline (nmol/g)	L-Glutamic acid (nmol/g)
CK	0.29±0.12b	1.86±0.60b	3878.50±189.38b	72.11±6.42b	39.81±2.18ab	225.62±16.03b	2817.86±195.32b	5829.42±384.22b	41.52±3.60b	30.21±2.69b	34.28±1.45b	195.41±18.47c
GO25	0.56±0.02a	3.30±0.28a	6668.06±78.44a	141.43±8.42a	31.70±4.37b	278.78±8.15a	5313.06±46.25a	11311.60±43.01a	116.71±2.65a	126.20±2.91a	80.76±2.33a	622.00±18.94a
BR	0.28±0.12b	1.73±0.44b	3477.04±138.67c	63.40±4.43a	41.31±5.77a	176.34±15.73c	2824.90±182.51b	7352.13±203.07c	40.91±2.14b	61.69±3.44b	22.25±1.80c	478.57±40.33b

Treatment	L-Glutamine (nmol/g)	L-Histidine (nmol/g)	L-Lysine (nmol/g)	L-Methionine (nmol/g)	L-Ornithine (nmol/g)	L-Phenylalanine (nmol/g)	L-Proline (nmol/g)	L-Threonine (nmol/g)	L-Tryptophan （nmol/g）	L-Tyrosine (nmol/g)	L-Valine (nmol/g)	Serine (nmol/g)
CK	542.64±31.72b	307.53±14.77b	33.72±1.56b	27.53±1.97b	133.67±10.02b	82.92±4.82c	1875.78±146.16b	651.79±34.01b	34.82±1.26b	44.12±2.20b	448.15±20.78b	329.61±20.32b
GO25	3167.18±111.20a	521.35±6.68a	46.46±1.23a	35.43±0.30a	188.69±1.69a	185.71±4.18a	3300.85±36.75a	929.77±11.82a	62.25±1.56a	58.40±1.98a	636.95±8.01a	719.79±7.34a
BR	614.12±47.88b	227.35±8.43c	39.24±2.23b	21.67±1.19c	92.62±5.90c	116.12±5.37b	1621.68±111.54c	628.73±47.25b	34.90±1.65b	47.71±2.77b	394.98±18.14c	347.74±21.11b

Note: Data are presented as mean±SD, CK refers to the control group; GO25 refers to the treatment with 25 mg/L of graphene oxide; BR refers to Bayer fertilizer. Data were analyzed by one-way ANOVA, Tukey's HSD test, n=3, with different letters indicating significant differences.
